# Preparation, optimization and in vitro–in vivo evaluation of Shunxin sustained release granules

**DOI:** 10.1186/s13020-019-0255-8

**Published:** 2019-09-23

**Authors:** Yinghuan Dou, Xuefeng Li, Yanbin Shi, Jiaying Zhang, Yang Yuan, Mengru Zhou, Xiangxiang Wei, Xiaoying Zhang

**Affiliations:** School of Basic Medical Sciences, Lanzhou University, Chengguan District, Donggang West Road No.199, Lanzhou, 730000 China

**Keywords:** Shunxin sustained release granules, Formula optimization, Drug release, Pharmacokinetics

## Abstract

**Background:**

Shunxinzufang decoction is tutors, empirical formula and has been used in Chinese patients of HFpEF for several years. The aim of this study was to make into sustained release granules and select the best formula for the preparation of Shunxin sustained release granules and to evaluate its in vivo and in vitro drug release behavior.

**Methods:**

Response surface methodology and Center composite design were applied to screen the optimal formula of Shunxin sustained release granules. HPLC was used to detect indicative ingredients—paeoniflorin, calycosin-7-glucoside and ferulic acid in Shunxin sustained release granules. The in vitro sustained release character of indicative ingredients was investigated in simulated digestive fluids. In-vivo process of active components was studied through pharmacokinetics.

**Results:**

The optimal formula of Shunxin sustained release granules consisted of 35% shunxinzufang extract and 65% HPMC/starch (HPMC/starch ratio = 2:1). Three indicative components can be separated well under selected HPLC conditions. Compared with Shunxinzufang extract, the active components of Shunxin sustained release granules have obvious sustained-release character and improved bioavailability.

**Conclusion:**

Shunxin sustained release granules has obvious sustained-release character and improved bioavailability.
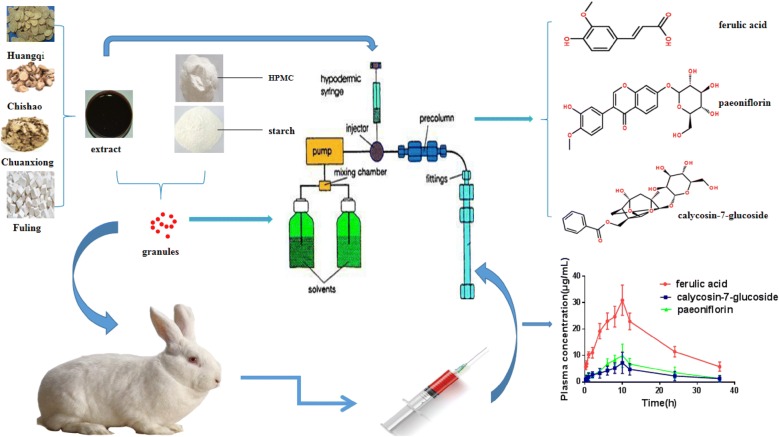

## Background

Heart failure with preserved ejection fraction (HFpEF) accounts for about 50% of patients with heart failure, and its only effective western medicine is diuretic [1]. Therefore, it is essential to find new therapeutics to overcome known chemicals’ disadvantages mentioned above or as prospective alternatives for the treatment of HFpEF. Generally, HFpEF patients requires long-term treatment of integrated traditional Chinese and western medicine.

In traditional Chinese medicine preparations, decoction is used to treat urgent diseases while pills or other solid dose forms are used to treat chronic diseases. Up to date, few of studies on sustained release preparations of Chinese medicine have been reported in Chinese or English periodicals. For example, Tongfeng granules, Yi-Shen granules, YuPing granules [2–4]. However, most of them are focusing on their pharmacodynamics, but few reported their formulation design, preparation and pharmacokinetics.

Shunxinzufang decoction consisted of twelve herbs: *Salviae Miltiorrhizae Radix Et Rhizoma* (Danshen)*, Radix ginsen Grubra* (Hongshen), *Astragali Radix* (Huangqi), *Radix Paeonia Rubra* (Chishao), Chuanxiong *Rhizoma* (Chuanxiong), *Poria* (Fuling), *Glycyrrhiza Radix Et Rhizoma* (Gancao) et al., which has been used in Chinese patients of HFpEF for several years. Although the decoction shows satisfied efficacy in clinical practice, it is unstable, not suitable for long-term storage and inconvenient to transport. In order to provide a stable and portable Chinese materia medica preparation for courses of treatment to patients with HFpEF, the shunxinzufang decoction was made into sustained release granules. Beside the above-mentioned advantages, the sustained release granules can achieve sustained release and stable blood drug concentration, improved bioavailability and further improve clinical efficacy. Compared with decoction, solid granules can avoid bitter taste and then improve the compliance of patients.

In this study, high performance liquid chromatography (HPLC) method was applied to simultaneously determine three indicative ingredients which were thought as main active compounds of Shunxin sustained-release granules to the treatment of HFpEF. Response surface methodology and central composite design (CCD) were adopted to further optimize the formula of Shunxin granules. The in vitro drug release and in vivo pharmacokinetics in rabbits were respectively investigated for optimized formula to validate its sustained release property.

## Methods

### Instruments and reagents

LC-10 ATVP high performance liquid chromatography equipped with a C18 chromatographic column and SLD-10AVP UV detector (Shimadzu, Japan), DGG-9620A thermostatic blast oven (Shanghai, China), Re-52AA rotary evaporator (Shanghai, China).

Chinese materia medica were all provided by Baibao Pharmaceutical company (Gansu, China). They were authenticated by Dr. Jianying Li, School of Pharmacy Lanzhou University. Hydroxy-propyl methyl cellulose (HPMC) and pharmaceutical starch were supplied by Shanghai Yuanye Biological Technology Co., Ltd. (Shanghai, China). Standard samples of paeoniflorin and Ferulic acid were purchased from Shanghai Yuanye Biological Technology Co., Ltd. (Shanghai, China). Calycosin-7-glucoside was obtained from China’s Food and Drug Verification Research Institute (Beijing, China). Chromatographic grade methanol was purchased from Fisher Scientific (Shanghai, China). Chromatographic grade acetonitrile was purchased from MREDA Technology Inc. (Beijing, China). All other reagents were analytical grade.

### Extraction of shunxinzufang extract and preliminary screening of excipient prescription

#### Preparation of shunxinzufang extracts and sustained release granules

Chinese materia medicas except *Radix ginseng rubra* are mixed together and firstly extracted with 8 times the volume of water for 2 h, then extracted with 4 times the amount of water twice and, each one took 1 h, respectively. *Radix ginsen Grubra* was singly extracted with 10 times the amount of water twice, and extracted with 8 times the amount of water for the third time. The water extracts are mixed together, filtrated and divided the Chinese materia medica solution equally. The filtrate was concentrated at 60 °C until the volume of extract sample was equivalent to the total quality of the tested Chinese materia medica. Then ethanol was added to the concentrated extract to precipitate polysaccharides. The resultant supernatant was transferred into a rotary evaporator to recover ethanol. Finally, the residue was evenly mixed with excipients, sifted through 16 mesh to make granules, dried at 60 °C, Shunxin granules was prepared.

#### Determination of the indicative ingredients of paeoniflorin, calycosin-7-glucoside and ferulic acid

The HPLC-system was equipped with a Diamonsil C18 (2) column (200 × 4.5 mm, 5 μm). The mobile phase consisted of acetonitrile and 0.1% phosphoric acid (17:83, v/v) with flow rate of 1.0 mL/min. The detection wavelength was set at 230 nm. The column temperature was 30 °C. The injection volume was 10 μL. A series of standard solutions of three indicative components including paeoniflorin, calycosin-7-glucoside and ferulic acid were made to construct standards curve. The receivable of the method was evaluated.

Preliminary screening of excipient

1. Filler screening
  Dextrin, lactose and starch are commonly used as fillers for Chinese materia medica granules. They are separately applied to make granules with matrix materials, and the optimal one was selected based on the granule formability, granule uniformity, fine powder rate and fluidity (angle of repose) as evaluation indexes.
2. Retardant screening
  Hydroxypropyl methyl cellulose (HPMC), sodium carboxymethyl cellulose (CMC-Na), microcrystalline cellulose (MCC), sodium alginate and carbopol were used as retardant for granulation. The evaluation indexes were the same mentioned above.

#### Single factor tests

When preparing a sustained-release preparation, hydrophilic but low solubility pharmaceutical materials are generally used, so starch was taken as filler, and HPMC as sustained-release material.

1. Extract content
  The mass ratio of HPMC and starch was fixed. Different amounts of extract were individually mixed with excipients to make granules. The granule formability, uniformity and fine powder rate were used as evaluation indexes. The extract content in the formula was screened out.
2. Ratio of HPMC and starch
  HPMC and starch mixed at a mass ratio of 1:1, 2:1, 3:1, 4:1 were used to make granules with fixed mass ratio of extract to excipient. The evaluation indexes were the same mentioned above.

### In vitro release test

Dissolution test was performed using RC-8D Dissolution tester (Tianjin Xintianguang company, China). The 2 g sustained-release granules were added into 900 mL purified water at 37 ± 0.5 °C. The speed of rotation was set at 100 r/min. At different time intervals (2, 4, 6, 8, 10 and 12 h), 1 mL samples was withdrawn and quickly supplemented by fresh resolution medium. The withdrawn samples subsequently were filtered through a 0.45 μm organic filtration membrane. 20 μL of filtrates were injected into HPLC for analysis.

### Central composite design experiment

A 2-factor, 5-level Central Composite design was used to optimize the Shunxin granule formula. According to the results of the single-factor tests, Shunxinzufang extract (%) (X_1_), HPMC/starch mass ratio (X_2_) were chosen as the main factors. The chosen dependent responses (Y_1_, Y_2_, Y_3_, Y_4_, Y_5_, Y_6_, Y_7_, Y_8_, Y_9_) were the cumulative release of Paeoniflorin, Calycosin-7-glucoside and Ferulic acid in 2 h, 6 h, 12 h. The independent variables (X_1_ and X_2_) and the responses in the experimental design were shown in Table 1. Experiments were automatically designed by Design Expert software (Version 8.0.6).

**Table 1:** Variables in central composite design experiments

Variables	Levels				
Independent variables	− α	− 1	0	+ 1	+ α
X_1_: shunxinzufang extract (%)	30.76	32	35	38	39.24
X_2_: HPMC/starch ratio	0.59:1	1:1	2:1	3:1	3.41:1

Dependent variables	Response range
Y_1_,Y_4_,Y_7_ (%)	Minimize
Y_2_,Y_5_,Y_8_ (%)	Target = 50
Y_3_,Y_6_,Y_9_ (%)	Maximize

Y_1_, Y_2_ and Y_3_ represent cumulative release of paeoniflorin at 2 h, 6 h, 12 h (%); Y_4_, Y_5_ and Y_6_ represent cumulative release of calycosin-7-glucoside at 2 h, 6 h, 12 h (%); Y_7_, Y_8_ and Y_9_ represent cumulative release of ferulic acid at 2 h, 6 h, 12 h (%)

### Pharmacokinetic study

#### Animal experiment

Six male New Zealand rabbits were divided into two groups with a weight range of 2.0–2.5 kg. The use of animals was approved by Animal Ethics Committee of School of Basic Medical Sciences of Lanzhou University. The rabbits were Fasted for 12 h before the experiment, but access to drinking water freely. The experimental rabbits were given single dose of 4.4 g/kg of Shunxin sustained release granules and control rabbits were given single dose of 2 g/kg of Shunxinzufang extract by oral administration.

Blood samples (about 2 mL) were collected into heparinized polyethylene tubes from heart before and after administration at 0.25, 0.5, 1, 2, 4, 6, 8, 10, 12, 24 and 36 h, respectively. The blood samples were processed by centrifugation at 1900×*g* for 10 min. Then, the supernatant was decanted into polypropylene tubes and stored at − 80 °C until analysis.

#### Plasma sample preparation

The plasma was naturally thawed at room temperature. Each 1.0 mL plasma sample was pipetted in a 10 mL centrifuge tube. 2.0 mL chromatographic methanol and 1.0 mL hydrochloric acid were added into plasma sample. 20 μL of hesperidin (IS), 4 mL ethyl acetate: iso-propyl alcohol (v:v, 1:1) and 1 mL of blank plasma were added, and vortexed for 2 min. Then, the mixture was centrifuged at 13,000×*g* for 10 min. The supernatant was transferred into autosampler vials for HPLC analysis. The mobile phase consisted of acetonitrile (A) and 0.1% phosphoric acid aqueous (B) (0–10 min 17% A, 10–15 min 35% A, 15–20 min 50% A, 20–25 min 35% A, 25–30 min 17% A). Pharmacokinetic parameters were calculated using DAS 2.1 Pharmacokinetic Software Package (Mathematical Pharmacology Professional Committee of China, Shanghai, China).

#### Validation of analytical method

Selectivity was evaluated by comparing the chromatography of blank plasma sample, plasma sample spiked with paeoniflorin, calycosin-7-glucoside, ferulic acid and internal standard (hesperidin), plasma sample obtained from tested rabbits, and Shunxin sustained release granules. The linear regression equations of 3 major active ingredients were conducted by plotting the peak area ratio (Y) of the components to IS versus the concentration (X) of components, respectively. The intra-day and inter-day precision and accuracy were determined by analyzing quality control (QC) samples containing three concentrations (low, middle and high) of three standards on the same day and on different days. The precision and accuracy were evaluated by determining the 3 major active ingredients in QC samples at three different concentrations. Extraction recoveries of 3 major active ingredients were calculated by comparing the peak area of the extracted QC samples with those of unextracted standard solution containing the equivalent amount of 3 major active ingredients. Stabilities of 3 components were evaluated by QC samples kept at − 20 °C for 7 days and after three freeze/thaw cycles (− 20 °C to room temperature as one cycle).

## Results

### In vitro HPLC analysis of three indicative components

**Fig. 1:**
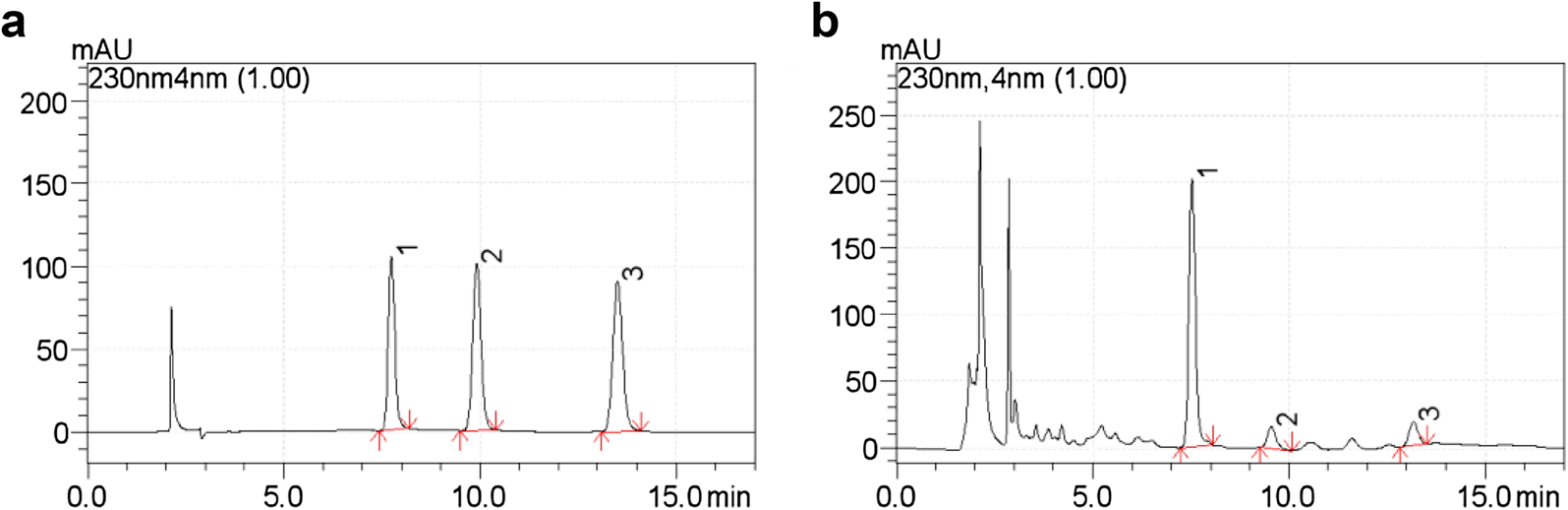
Chromatograms of the standard solution (**a**), the test solution (**b**); 1, 2, and 3 represent paeoniflorin, calycosin-7-glucoside and ferulic acid, respectively

Chromatographic peaks of paeoniflorin (1), calycosin-7-glucoside (2), and ferulic acid (3) can be well separated in shunxinzufang extracts (Fig. 1). The standard curve is drawn with the peak area as the ordinate (Y) and the mass concentration as the abscissa (X). The regression equations are Y = 12403x + 1555.6, (R^2^ = 0.9999), Y = 29700x + 449.7, (R^2^ = 1), Y = 31333x − 26,615, (R^2^ = 0.9999), indicating that paeoniflorin, calycosin-7-glucoside and ferulic acid have a good linear relationship. The average recoveries of paeoniflorin, calycosin-7-glucoside and ferulic acid were 98.3%, 90.5%, and 97.6%, respectively, and the RSD was 0.87%, 0.87%, and 0.50%, respectively. The details are listed in Additional file 1: Table S1.

### Filler and retardant

1. Filler
  It can be seen from Table 2 that the fine powder rate of dextrin filled granules was relatively higher compared with the other two fillers. After being wetted with water, it was of strong viscosity and was not easy to be sieved and granulated. Although lactose has good moisture resistance, poor moldability, but it is also expensive, and not suitable for large-scale use. The starch is cheap, biodegradable and biocompatible, and does not react with biologically active drugs. Which is the most commonly used pharmaceutical excipients. Taking these factors into account, starch was adopted to make granules.
2. Retardants
  Table 3 listed the screening results of the three matrix materials. During the granulation process, CMC-Na granules showed serious agglomeration, poor particle uniformity, and many fine powders. Both granules made with MCC and HPMC were satisfied, but MCC is relatively expensive. And considering the different pH of different parts of the gastrointestinal tract, HPMC, a non-pH dependent hydrogel material, was the ideal retardants. HPMC has good sustained-release ability for many different types of drugs, and is often used as a hydrophilic gel material for controlled release formulations [5]. A small amount of HPMC also has a certain viscosity when it is in contact with water, which is not conducive to sieve. However, starch can reduce the viscosity of HPMC. Therefore, the compatibility of starch and HPMC would result in a reasonable viscosity of mixture.

**Table 2:** Filler screening

Species	Formability	Homogeneity	Fine powder rate (%)	Angle of repose (°)
Starch	Easy, slight adhesion to sieve	+	4.94	30.99 ± 1.08
Dextrin	Easier, easy to adhere to sieve	+	6.42	31.68 ± 1.97
Lactose	Easy, slight adhesion to sieve	+	5.19	32.99 ± 1.20

+, satisfied; −, not satisfied

**Table 3:** Matrix material screening

Species	Formability	Homogeneity	Fine powder rate (%)	Angle of repose (°)
HPMC	Easier, slight adhesion to sieve	+	4.94	30.99 ± 1.08
CMC-Na	Easier, agglomeration	+	8.28	37.78 ± 1.52
MCC	Easier, slight adhesion to sieve	+	5.22	29.81 ± 1.77

+, satisfied; −, not satisfied

### Single factor test

**Fig. 2:**
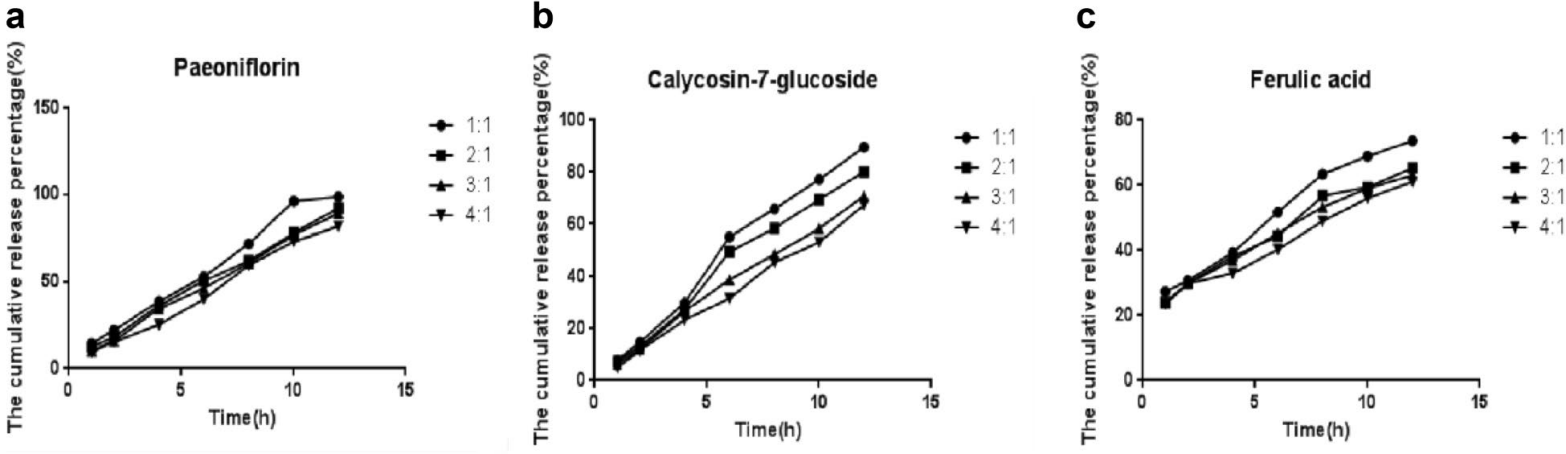
Cumulative release percentage

1. Extract ratio
  As can be seen from the Table 4, it is reasonable that the extract mass ratio accounted for 32–38% of the total formula. Therefore, in the subsequent experimental design, the extract content is selected from 32 to 38%.
2. HPMC and starch mass ratio
  It can be seen from Fig. 2 that when the mass ratio is 4:1, the three components cannot be completely released. At ratios of 1:1, 2:1, 3:1, the three components can be released relatively well.

**Table 4:** Extract ratio screening

Extract ratio	Formability	Homogeneity	Fine powder rate (%)	Angle of repose (°)
30%	Easy	+	−	21.05
32%	Easy	+	+	8.49
35%	Easier, slight adhesion to sieve	+	+	5.15
38%	Easier, adhesion to sieve	+	+	4.94

+, satisfied; −, not satisfied

### Central composite design statistical analysis

**Fig. 3:**
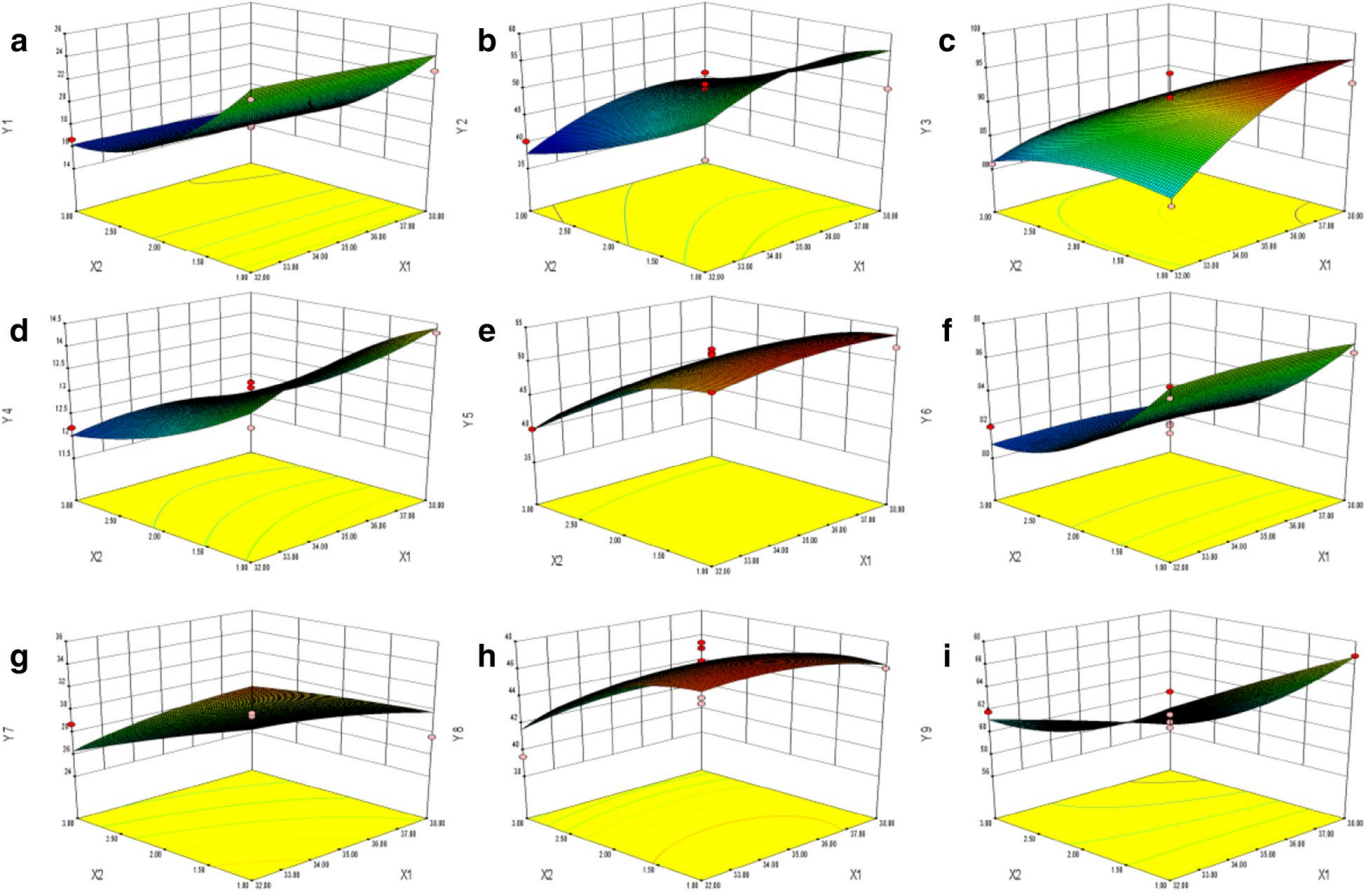
Three-dimensional effect surface map. Y_1_, Y_2_ and Y_3_ represent cumulative release of paeoniflorin at 2 h, 6 h, 12 h (%) (**a**, **b**, **c**); Y_4_, Y_5_ and Y_6_ represent cumulative release of calycosin-7-glucoside at 2 h, 6 h, 12 h (%) (**d**, **e**, **f**); Y_7_, Y_8_ and Y_9_ represent cumulative release of ferulic acid at 2 h, 6 h, 12 h (%) (**g**, **h**, **i**)

The Central Composite design results, using Design-Expert.v8.0.6. software to draw a three-dimensional effect surface map of the influence of various factors on the effect value (Fig. 3). Combining the three-dimensional effect surface map, using the Design-Expert.v8.0.6. software to predict the regression equation, the optimized formulation with the observed and predicted response values consisted of 35.06% shunxinzufang extract (X_1_) and 2.38:1 HPMC/starch ratio (X_2_). As expected, the result experimental values was in agreement with the values calculated from the models. Therefore, it can be concluded that response surface methodology is an effective method to optimize formulation of Shunxin sustained release granules. From Fig. 3, at 2 h and 6 h, X_1_ has no significant effect on the cumulative release rate of paeoniflorin, but slightly increased at 12 h. The cumulative release rate of paeoniflorin in X_2_ increased first and then decreased. At 2 h and 6 h, with the increase of X_1_, the cumulative release rate of calycosin-7-glucoside increased slightly, but was not significant at 12 h. X_2_ showed a trend of increased, decreased and then increased on the accumulation release rate of calycosin-7-glucoside. At 2 h and 6 h, X_1_ had no significant effect on the cumulation release rate of ferulic acid. At 12 h, it increased with the increase of X_1_. The cumulative release rate of ferulic acid by X_2_ increased first and then decreased. In summary, X_2_ significantly affected the cumulative release percentage of paeoniflorin, calycosin-7-glucoside and ferulic acid.

The in vitro cumulative release rates of paeoniflorin, calycosin-7-glucoside and ferulic acid at 2 h, 6 h and 12 h were 17.58%, 52.05%, 89.15%; 14.28%, 48.05%, 84.15%; 29.43%, 46.93%, 62.65%, respectively, which indicated the mathematical model obtained by Design-Expert.v8.0.6. software was reliable and good for predictability. The details are listed in Additional file 1: Tables S2, S3, S4.

### Pharmacokinetic analysis

**Fig. 4:**
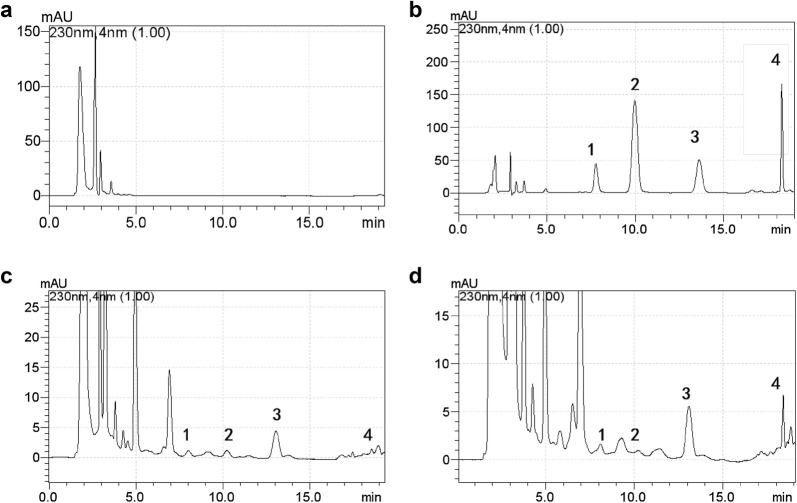
Typical chromatograms of plasma samples. Chromatograms of blank plasma (**a**), blank plasma spiked with the analytes and IS (**b**), a plasma sample after oral administration of Shunxinzufang extract (**c**) and a plasma sample after oral administration of Shunxin sustained release granules (**d**). 1, 2, 3 and 4 represent paeoniflorin, calycosin-7-glucoside, ferulic acid and IS, respectively

Figure 4 shows the HPLC chromatograms of blank plasma (A), blank plasma spiked with IS (B), a plasma sample at 6 h after oral administration of Shunxinzufang extract (C) and a plasma sample at 10 h after oral administration of Shunxin sustained release granules (D). All target compounds were effectively separated. Typical equations for the calibration curves and correlation coefficients (R^2^) were as follows: Y = 0.1237x + 0.2419(R^2^ = 0.9993) for paeoniflorin, Y = 0.2621x + 0.209(R^2^ = 0.9983) for calycosin-7-glucoside, Y = 0.2381x − 0.7562(R^2^ = 0.9974) for ferulic acid. The calibration curves of the three analytes were linear in the ranges of 0.2–400 μg/mL for paeoniflorin, 0.1–200 μg/mL for calycosin-7-glucoside, 0.5–200 μg/mL for ferulic acid.

**Fig. 5:**
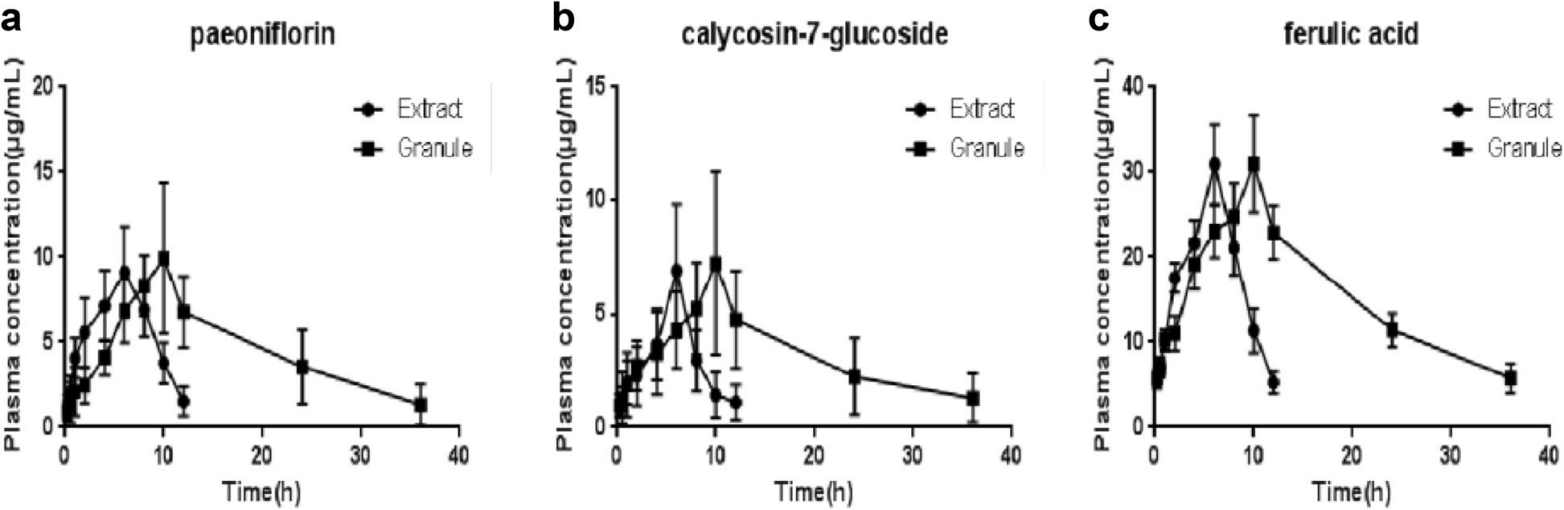
Plasma concentration–time curves of the three compounds

The results of the precision, accuracy, extract recovery and stability at three concentration levels are summarized in Table 5. The plasma concentration–time curves of the three active components are shown in Fig. 5. Statistical analysis of the pharmacokinetic parameters (t_1/2_, T_max_, C_max_, AUC (0 − t), AUC (0 − ∞), MRT (0 − t), MRT (0 − ∞)) are shown in Table 6.

**Table 5:** The accuracy (intra- and inter-day) (n = 5), precision (n = 5), extract recovery (n = 5) and stability (n = 3) for 3 components in rabbits plasma

Components	Concentration (μg/mL)	Accuracy (RSD%)		Precision (%)	Extract recovery (%)	Stability (RSD%)	
		Inter-day	Intra-day			At − 20 °C 7 days	Freeze–thaw cycles
Paeoniflorin	Low	5.68	4.13	102.03	89.68	5.52	6.15
	Medium	2.47	2.78	103.47	94.07	3.37	6.90
	High	3.36	6.31	98.79	95.99	4.17	8.25
Calycosin-7-glucoside	Low	4.84	7.25	105.68	86.90	5.28	7.90
	Medium	4.08	5.57	88.53	89.91	5.57	6.53
	High	6.33	7.44	98.33	91.33	5.69	7.14
Ferulic acid	Low	2.38	7.84	86.56	79.90	3.24	8.09
	Medium	3.10	2.24	96.05	82.08	4.24	6.87
	High	5.68	8.76	97.26	84.66	7.09	8.40

**Table 6:** Pharmacokinetic parameters

	Paeoniflorin		Calycosin-7-glucoside		Ferulic acid	
	Extract	Granule	Extract	Granule	Extract	Granule
C_max_ (μg/mL)	9.32 ± 2.47	11.37 ± 2.30	6.90 ± 2.93	8.89 ± 2.15	3.90 ± 4.68	32.37 ± 4.10
T_max_ (h)	6 ± 1.63	9.5 ± 1.00	6 ± 0.00	10 ± 1.63	6.5 ± 1.00	9.5 ± 1.00
AUC (0 − t) (μg/mL h)	67.99 ± 10.89	162.72 ± 49.11	36.58 ± 13.81	114.07 ± 29.92	215.41 ± 22.43	553.17 ± 52.30
AUC (0 − ∞) (μg/mL h)	73.54 ± 7.39	190.71 ± 86.77	37.62 ± 13.84	146.20 ± 60.82	232.18 ± 15.73	659.68 ± 82.83
MRT (0 − t) (h)	5.80 ± 0.32	14.12 ± 1.98	5.76 ± 0.18	13.85 ± 2.89	5.82 ± 0.15	14.33 ± 0.25
MRT (0 − ∞) (h)	6.57 ± 0.87	18.64 ± 9.17	6.02 ± 0.22	21.86 ± 11.03	6.53 ± 0.45	20.72 ± 3.24
t_1/2_ (h)	2.10 ± 0.80	9.47 ± 6.92	1.55 ± 0.49	12.89 ± 7.00	2.04 ± 0.72	12.10 ± 3.13

From Fig. 5 and Table 6, we can know that the Shunxin sustained release granules have obvious sustain-release character compared with Shunxinzufang extract. Shunxinzufang extract is a liquid preparation, and its absorption is faster than that of the sustained-release granules, which is reflected in the peak time (6 h and 10 h respectively), indicating that the granules has sustained-release character. From Table 6, it can be seen that the biological half-life (t_1/2_) of Shunxin sustained release granules is significantly longer than Shunxin extract; the mean residence time [MRT (0 − t) and MRT (0 − ∞)] is significantly longer than the latter. These results confirmed that the sustained release granules have obvious release characteristics of paeoniflorin, calycosin-7-glucoside and ferulic acid. The area under the curve (AUC) of the sustained release granules was also significantly higher than the extract, demonstrating that the former preparation has higher bioavailability.

## Discussion

Chinese medicine has a reliable therapeutic effect on chronic heart failure, which can significantly improve the long-term prognosis of these patients and improve the quality of life. However, patients need to take long-term medication. Decoction is the main dosage form in the TCM clinical practice, but it is not stable, and not suitable for long-term storage. Thus Chinese materia medica needs to be freshly prepared and preserved for not more than 24 h, which reduces the compliance of patients with multi-courses treatment. The sustained and controlled release preparations can not only extend intervals between doses by slow release of active components, but also decrease possible systemic side-effect by maintaining relatively stable blood concentration after repeated medication. Shunxinzufang has been efficaciously used in patients of HFpEF. Among them, *Astragali Radix* has efficacies of tonifying qi and lifting yang, stopping perspiration with strengthening superficies, subsidening swelling and diuresis et al. Morden pharmacological research verified it has immune-stimulant, anti-bacterial, antiviral, hepatoprotective, anti-inflammatory, cardiovascular protection, and vasodilatory action [6–9]. Calycosin-7-glucoside, as the main component, has anti-oxidant, anti-inflammatory activities [10, 11]. *R*Chuanxiong *Rhizoma* has the effect of promoting blood circulation, activating qi and relieving pain. It has antioxidant [12, 13], neuroprotection [14, 15], anti-inflammatory [16], anti-proliferative [17], anti-proliferation [18], and proapoptotic activities [19]. One of its main chemical constituent, ferulic acid, has anti-inflammatory, anti-oxidative and anti-tumor activity [20–22]. *Radix Paeonia Rubra* has effects of removing heat to cooling blood, promoting blood circulation to remove blood stasis. It has immuno-regulatory, anti-oxidant, antiallergic, and anti-inflammatory effects [23–25]. Paeoniflorin, the main compound isolated from its root, has anti-inflammatory, antioxidant, antidepressant and apoptotic effects [23, 26–28]. Taking the above-mentioned together, Shunxin sustained release granules have been designed and prepared, and its in vitro release property and in vivo pharmacokinetics with paeoniflorin, calycosin-7-glucoside and ferulic acid as the indicative components have also investigated.

HPMC has been reported showing an ideal function of extending drug release in drug delivery systems [29, 30]. Phaechawud et al. pointed that drug release was prominently sustained for over 12 h using HPMC-based hydrophilic matrix system [31]. Corn starch showed an increase in flowability with increasing moisture content, and the flowability of corn starch increased with moisture content until monolayer coverage was reached as the water provided increasing interparticle lubrication [32]. In this study, HPMC was used as the main sustained-release material and starch as the filling agent to complete the sustained-release characteristics of the particles. Response surface methodology and CCD were applied to screen the optimal formula of Shunxin sustained release granules. The optimal formula of Shunxin sustained release granules was 35% shunxinzufang extract and 65% HPMC/starch (HPMC/starch ratio = 2:1, w/w). When HPMC was used as the retardant material, it had a certain viscosity when exposed to water, while dried starch lack of strong viscosity. Therefore, starch was added in the granulation process to reduce the viscosity of HPMC. The details are listed in Additional file 1: Fig. S1.

In preliminary tests, it was found that the Shunxin sustained release granules can release about 62–90% marker components, which basically satisfy the requirement of sustained release preparations in China Pharmacopoiea [33]. Generally, sustained release preparations were administrated once daily or 2 times a day. After comprehensive consideration, we choose 12 h to take dissolution samples for analyses.

The cumulative release rate of paeoniflorin and calycosin-7-glucoside in 12 h were 89.15%, 84.15%, and ferulic acid was relatively low (62.65%). The release mechanism may be related to the characteristics of HPMC, mainly because of the three components in sustained release granules concentration difference inside and outside. And the concentration difference is used as the main driving force to release the paeoniflorin, calycosin-7-glucoside, and ferulic acid through the HPMC in granules surface formation of the gel layer and slow release. Three components have unreleased fully within 12 h, for the three components have not been completely released under the gel layer formed by HPMC. The results of pharmacokinetics showed that Shunxin sustained release granules had obvious sustained release characteristics and could significantly improve their bioavailability. The correlation coefficient squares (R^2^) between in vitro release and in vivo absorption for paeoniflorin, Calycosin-7-glucoside and ferulic acid were 0.9386, 0.9506 and 0.9431, respectively. These data showed the drug release in vitro and their absorption in rabbits contained good relativity, further proving that the optimized shunxin granules realized the purpose of this study.

Under the conditions of this formula, Shunxin sustained release granules demonstrated the advantages of stable quality, easy storage, convenient carrying and taking, etc. And it would create favorable conditions for improving medication compliance in patients with heart failure and further improving clinical efficacy. As adjuvant drugs, to combine with conventional chemical drugs, may be more effective for HFpEF. At present, there was not seen the application of sustained and controlled release preparations of Chinese materia medica on clinical practice in China. However, the whole controlled release of multi-components of Chinese materia medica is a major difficulty in the study of Chinese herbs sustained and controlled release preparations, and it is also the direction for further improvement of the preparation technology of this experiment.

## Conclusion

Shunxin sustained release granules has obvious sustained-release character and improved bioavailability.

## Supplementary information

**Additional file 1.** Other data tables and figure.

## Data Availability

All data are fully available without restriction.

## Abbreviations

HFpEF: heart failure with preserved ejection fraction
HPLC: high performance liquid chromatography
HPMC: hydroxypropyl methyl cellulose
CMC-Na: sodium carboxymethyl cellulose
MCC: microcrystalline cellulose
QC: quality control
