# Supplementary material for: Preparation, optimization and in vitro–in vivo evaluation of Shunxin sustained release granules
Source: Chin Med. 2019 Sep 23;14:36. doi: 10.1186/s13020-019-0255-8 (PMC6757358; doi:10.1186/s13020-019-0255-8)
Supplement: Supplementary file 1 — Additional file 1. Other data tables and figure. [file 13020_2019_255_MOESM1_ESM.pdf]

Table S1 Accuracy, Precision, Stability, Sample recovery（n=6）

Tests	paeoniflorin	calycosin-7-glucoside	ferulic acid
Accuracy（RSD）	0.13%	0.55%	0.30%
Stability（RSD）	0.47%	0.58%	1.9%
Precision（RSD）	0.45%	0.38%	0.68%
Sample recovery (average recovery）	98.3%	90.5%	97.6%
Sample recovery（RSD）	0.87%	0.87%	0.50%

Table S2 Test design and results

run	X_1 （%）	X_2 （%）	Y_1 （%）	Y_2 （%）	Y_3 （%）	Y_4 （%）	Y_5 （%）	Y_6 （%）	Y_7 （%）	Y_8 （%）	Y_9 （%）
1	35	0.59:1	29.98	69.97	94.59	15.22	54.23	90.79	37.08	47.35	69.92
2	38	1:1	22.80	50.03	92.76	14.29	52.09	86.31	27.59	46.06	66.73
3	35	2:1	18.52	50.88	89.31	13.10	51.33	82.00	29.94	47.91	61.01
4	39.24	2:1	18.31	50.66	94.13	12.57	52.47	81.66	26.56	45.95	59.02
5	32	3:1	16.68	40.20	80.77	12.20	40.06	81.92	28.75	39.50	61.84
6	35	3.41:1	16.2	39.82	82.80	11.78	37.74	80.05	20.24	42.38	57.79
7	32	1:1	24.1	46.17	82.42	13.27	51.85	86.24	33.61	46.62	65.50
8	35	2:1	17.81	49.57	90.28	12.93	50.40	82.12	29.42	47.52	60.98
9	38	3:1	15.53	45.86	80.17	12.30	40.42	81.80	26.46	40.86	57.98
10	35	2:1	18.07	49.92	94.29	13.22	51.88	83.55	29.68	45.11	63.65
11	30.76	2:1	18.34	40.68	80.38	12.29	46.68	81.72	31.93	46.81	62.52
12	35	2:1	17.88	49.82	90.73	12.81	51.07	81.54	29.69	46.59	60.97
13	35	2:1	18.01	49.36	87.85	12.95	48.60	82.87	29.48	45.57	60.45

Y_1, Y_2 and Y_3 respresent cumulative release of paeoniflorin at 2h, 6h, 12h (%); Y_4, Y_5 and Y_6 respresent cumulative release of calycosin-7-glucoside at 2h, 6h, 12h (%); Y_7, Y_8 and Y_9 respresent cumulative release of ferulic acid at 2h, 6h, 12h (%).

Table S3 Fitting equation

Response	Correlation coefficient
$Y_1=26.77+0.64X_1-13.84X_2+0.01X_1X_2-0.01X_1^2+2.28X_2^2$	$R^2=0.9703$
$Y_2=-404.69+26.35X_1-17.1X_2+0.15X_1X_2-0.37X_1^2+1.31X_2^2$	$R^2=0.7408$
$Y_3=-370.23+23.3X_1+35.58X_2-0.91X_1X_2-0.29X_1^2-1.88X_2^2$	$R^2=0.8278$
$Y_4=-28.77+2.34X_1+0.65X_2-0.08X_1X_2-0.03X_1^2+0.26X_2^2$	$R^2=0.9425$
$Y_5=-91.35+7.81X_1+4.82X_2+1E\text{-}002X_1X_2-0.11X_1^2-2.75X_2^2$	$R^2=0.9463$
$Y_6=62.06+1.85X_1-8.97X_2-0.02X_1X_2-0.03X_1^2+1.63X_2^2$	$R^2=0.9191$
$Y_7=58.32-0.03X_1-12.79X_2+0.31X_1X_2-0.02X_1^2-0.45X_2^2$	$R^2=0.7869$
$Y_8=-33.81+4.88X_1-2.31X_2+0.16X_1X_2-0.07X_1^2-1.43X_2^2$	$R^2=0.7518$
$Y_9=35.88+1.67X_1+5.56X_2-0.42X_1X_2-0.02X_1^2+1.4X_2^2$	$R^2=0.9261$

Y_1, Y_2 and Y_3 respresent cumulative release of paeoniflorin at 2h, 6h, 12h (%); Y_4, Y_5 and Y_6 respresent cumulative release of calycosin-7-glucoside at 2h, 6h, 12h (%); Y_7, Y_8 and Y_9 respresent cumulative release of ferulic acid at 2h, 6h, 12h (%).

Table S4-1 ANOVA for responses

Source	Sum of Squares			df			*F* value			*P*-value prob>*F*		
	Y_1	Y_2	Y_3	Y_1	Y_2	Y_3	Y_1	Y_2	Y_3	Y_1	Y_2	Y_3
Model	184.19	516.07	319.94	5	5	5	45.71	4.00	6.73	<0.0001	0.049	0.013
X_1	0.78	69.82	106.47	1	1	1	0.96	2.71	11.2	0.3590	0.143	0.012
X_2	146.02	348.20	119.46	1	1	1	181.20	13.50	12.5	<0.0001	0.007	0.009
X_1X_2	5.625E-003	0.81	29.92	1	1	1	6.980E-003	0.031	3.15	0.9358	0.864	0.119
X_1^2	0.068	75.73	47.20	1	1	1	0.085	2.94	4.96	0.7796	0.130	0.061
X_2^2	36.27	12.00	24.71	1	1	1	45.01	0.47	2.60	0.0003	0.517	0.150
Residual	5.64	180.53	66.54	7	7	7	-	-	-	-	-	-
Lack of	5.33	179.17	43.64	3	3	3	23.00	174.7	2.54	0.0055	0.000	0.194
Pure Error	0.31	1.37	22.90	4	4	4	-	-	-	-	-	-
Cor Total	189.63	696.60	386.49	12	12	12	-	-	-		-	-

Y_1, Y_2 and Y_3 respresent cumulative release of paeoniflorin at 2h, 6h, 12h (%).

Table S4-2 ANOVA for responses

Source	Sum of Squares			df			F value			P-value prob>F		
	Y_4	Y_5	Y_6	Y_4	Y_5	Y_6	Y_4	Y_5	Y_6	Y_4	Y_5	Y_6
Model	9.49	338.53	92.03	5	5	5	22.93	24.65	15.91	0.0003	0.0003	0.0011
X_1	0.29	9.65	2.273E-003	1	1	1	3.47	3.51	1.965E-003	0.1048	0.1030	0.9659
X_2	7.85	273.55	72.11	1	1	1	94.81	99.59	62.32	<0.0001	<0.0001	<0.0001
X_1X_2	0.21	3.600E-003	9.025E-003	1	1	1	2.56	1.311E-003	7.800E-003	0.1539	0.9721	0.9321
X_1^2	0.52	6.40	0.38	1	1	1	6.28	2.33	0.33	0.0406	0.1707	0.5827
X_2^2	0.48	52.77	18.94	1	1	1	5.75	19.21	15.98	0.0477	0.0032	0.0052
Residual	0.58	19.23	8.10	7	7	7	-	-	-	-	-	-
Lack of Fit	0.48	12.81	5.58	3	3	3	6.25	2.66	2.95	0.0544	0.1839	0.1614
Pure Error	0.10	6.42	2.52	4	4	4	-	-	-	-	-	-
Cor Total	10.07	357.76	100.13	12	12	12	-	-	-	-	-	-

Y_4, Y_5 and Y_6 respresent cumulative release of calycosin-7-glucoside at 2h, 6h, 12h (%).

Table S4-3 ANOVA for responses

Source	Sum of Squares			df			*F* value			*P*-value prob>*F*		
	Y_7	Y_8	Y_9	Y_7	Y_8	Y_9	Y_7	Y_8	Y_9	Y_7	Y_8	Y_9
Model	147.65	63.59	137.24	5	5	5	5.17	4.24	17.56	0.0265	0.0429	0.0008
X_1	31.62	0.022	7.18	1	1	1	5.53	7.220E-003	4.59	0.0509	0.9347	0.0693
X_2	111.04	46.80	109.26	1	1	1	19.44	15.60	69.88	0.0031	0.0055	<0.0001
X_1X_2	3.48	0.92	6.48	1	1	1	0.61	0.31	4.14	0.4608	0.5966	0.0813
X_1^2	0.18	3.13	0.15	1	1	1	0.032	1.04	0.095	0.8640	0.3411	0.7671
X_2^2	1.43	14.19	13.57	1	1	1	0.25	4.73	8.68	0.6321	0.0661	0.0215
Residual	40.00	21.00	10.94	7	7	7	-	-	-	-	-	-
Lack of Fit	39.83	15.17	4.47	3	3	3	315.94	3.47	0.92	<0.0001	0.1302	0.5078
Pure Error	0.17	5.83	6.48	4	4	4	-	-	-	-	-	-
Cor Total	187.64	84.58	148.19	12	12	12	-	-	-	-	-	-

Y_7, Y_8 and Y_9 respresent cumulative release of ferulic acid at 2h, 6h, 12h (%).

Fig. S1

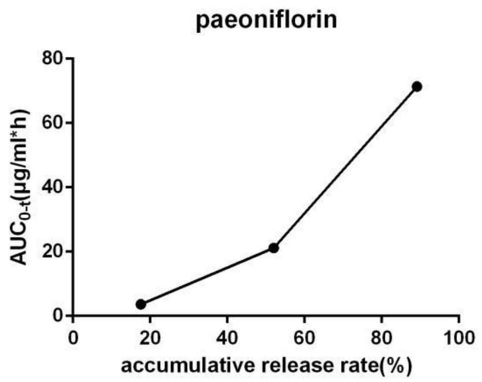

Y= 0.9521X - 18.331, R^2 = 0.9386

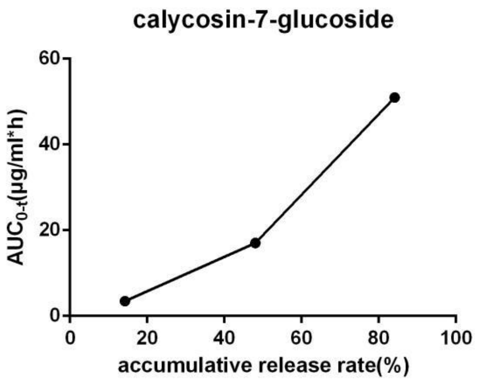

Y = 0.6828X - 9.4942, R^2 = 0.9506

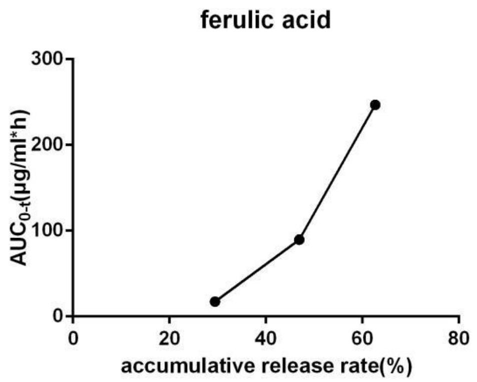

Y = 6.86X - 199.9, R^2 = 0.9431
